# Treating and Preventing No Reflow in the Cardiac Catheterization Laboratory

**DOI:** 10.2174/157340312803217148

**Published:** 2012-08

**Authors:** Ryan Berg, Cyrus Buhari

**Affiliations:** UCSF Fresno Division of Cardiology, 2823 Fresno Street, 5th Floor, Fresno, CA 93721

**Keywords:** No-reflow, adenosine, verapamil, nitroprusside, aspiration thrombectomy, distal protection.

## Abstract

The no reflow phenomenon can happen during elective or primary percutaneous coronary intervention. This phenomenon is thought to be a complex process involving multiple factors that eventually lead to microvascular obstruction and endothelial disruption. Key pathogenic components include distal atherothrombotic embolization, ischemic injury, reperfusion injury, and susceptibility of coronary microcirculation to injury. Thus, pharmacologic and mechanical strategies to prevent and treat no reflow target these mechanisms. Specifically, pharmacologic therapy consisting of vasodilators and antiplatelet agents have shown benefit in the treatment of no-reflow and mechanical therapies such as distal protection and aspiration thrombectomy have also shown benefit.

## BACKGROUND

The “no reflow phenomenon” has various definitions. Classically, it is considered to be the lack of myocardial perfusion despite opening up the epicardial vessel in the setting of primary percutaneous coronary intervention (PCI). It can occur in up to 10% of cases of primary PCI and is associated with an increased 30 day mortality if not adequately treated (32% vs. 2.8%, p<.0.001) [1]. In this setting, the phenomenon is thought to be a complex process involving multiple factors that eventually lead to microvascular obstruction and endothelial disruption. Key pathogenic components include distal atherothrombotic embolization, ischemic injury, reperfusion injury, and susceptibility of coronary microcirculation to injury. Thus, pharmacologic and mechanical strategies to treat no reflow target these mechanisms (see Figs. (**1** and **2**)). 

However, the “no reflow phenomenon” is sometimes taken broadly to mean sudden loss of epicardial flow (abrupt onset of thrombolysis in myocardial infarction (TIMI) zero flow, typically after ballooning a lesion or placement of a stent. In this setting, the “no-reflow phenomenon” can be secondary to microvasculature obstruction or dysfunction as described above, or it can be secondary to incomplete lesion dilation, epicardial spasm, or epicardial dissection with or without in situ thrombosis. The first step in management in these cases would be use of intravascular ultrasound to distinguish dissection and spasm from microvascular phenomenon. Treatment of epicardial spasm and dissection is outside the scope of this chapter. Rather we will review the current pharmacologic and mechanical treatment/management of no reflow in the catheterization lab with focus on microvasculature obstruction or dysfunction, in primary or elective PCI 

## PHARMACOLOGIC THERAPY

Pharmacotherapy for the treatment of no reflow has focused primarily on two strategies: local vasodilator therapy and local antiplatelet therapy. Of these, only local vasodilator therapy has a specific guideline indication for treatment of no-reflow. The 2011 ACC PCI guidelines [2] give a class IIa recommendation for administration of an intracoronary vasodilator (specifically, adenosine, calcium channel blocker, or nitroprusside) to treat PCI-related no-reflow that occurs during primary or elective PCI. 

### Adenosine

Adenosine has been widely studied for the treatment of no reflow. Among its many beneficial effects, adenosine increases microvascular flow owing to its vasodilator properties, inhibits neutrophil adhesion and migration, exerts antiplatelet effects, and inhibits oxygen free radical formation, which results in decreased cellular acidosis. [3]. Adenosine has shown benefit in both intravenous and intracoronary administration. 

*Intravenous *adenosine has been tested in two randomized trials (AMISTAD [4] and AMISTAD II [5]). The AMISTAD trial randomized 236 patients to thrombolysis (alteplase or streptokinase) with IV adenosine or placebo. The trial was designed to assess if adenosine as an adjunct to thrombolysis would reduce myocardial infarct size as measured by SPECT imaging. Stratification by myocardial infarction (MI) location was performed and then patients were randomly assigned to peripherally infused adenosine at 70 mcg/kg/min for three hours or placebo. The study drug was given before intiation of thrombolytic therapy. There was a 33% relative reduction in infarct size with adenosine using multivariate regression analysis (p=0.03) in all patients, and there was a more significant 67% relative reduction (p=0.014) in pateints with anterior MI. 

The AMISTAD II trial was a placebo controlled, phase III trial to compare the incidence of death and congestive heart failure (CHF) in acute myocardial infarction. Patients were randomized to placebo or one of two doses of IV adenosine (50mcg/kg/min or 70mcg/kg/min). This was an adjunct to either thrombolysis or angioplasty. 2118 patients were enrolled and randomized IV adenosine or placebo for 3 hours. Patients underwent reperfusion (thrombolysis or mechanical reperfusion) within 15 minutes after initiation of study drug infusion. The primary endpoint was new CHF, first rehospitalization for CHF or death from any cause at 6 months. There was no significant difference in the primary endpoint between the pooled adenosine groups and placebo. In retrospect the study was likely underpowered to appropriately look at this endpoint. However, in concordance with the data from the original AMISTAD trial, infarct size in the higher dose adenosine group (70mcg/kg/min – the same dose used in the AMISTAD trial) was significantly decreased when compared to placebo (11% vs 27%, p=0.023). 

*Intracoronary *adenosine has also shown benefit in preventing no reflow. It is more likely to see potential side effects of adenosine when given by the intracoronary route, especially in high doses. However, these same side effects can be seen in intravenous dosing. These side effects include flushing, hypotension, bradycardia with various degrees of heart block, dyspnea, chest pain, and bronchospasm. In a retrospective series, intracoronary adenosine (24-48mcg) was given before and after each balloon inflation in the setting of an acute myocardial infarction. No reflow was noted in 5.9% in the intracoronary adenosine group compared to 28.6% of controls (p=0.014) [6] In a smaller(n=54) randomized trial [3], 4mg of intracoronary adenosine was given over 1 minute through an over the wire balloon inflated at the culprit lesion as compared to a saline placebo. The no reflow phenomenon occurred in in 4% of the adenosine treated patients as compared to 26% of the control patients (p=.02). In a larger (n=448) randomized intracoronary adenosine trial, the ADAPT trial, there was no significant difference in the no-reflow phenomenon as assessed by myocardial blush and TIMI flow when comparing the adenosine group and the control group. However, there was also >90% mechanical aspiration and IIbIIIa use in this trial which likely diluted the expected result [7]. 

### Calcium Channel Blocker

Calcium channel blockers may improve coronary flow through endothelium-dependent and -independent relaxation. Calcium channel blockers work via several mechanisms including endothelium-mediated vasodilatation, reduction of myocardial oxygen demand by negative inotropic and chronotropic effects, and may reduce oxygen free radical damage during reperfusion. Verapamil has been the most studied calcium channel blocker for prevention and treatment of the no-reflow phenomenon. 

In a randomized trial of 149 patients undergoing PCI for acute coronary syndrome, patients received either a single intracoronary bolus of adenosine, verapamil, or saline after angioplasty. The primary endpoint was the change in TIMI frame counts after administration of the study drug. The improvement in TIMI frame counts showed statistical significance in verapamil or adenosine in STEMI patients as compared to the control saline group(8.5% vs. 0%). However, there was no significant difference seen between verapamil and adenosine. This is suggestive that verapamil can act as a preventative measure [8]. 

In a smaller prospective study, verapamil has also shown it can actively treat no-reflow after it has occurred. 212 consecutive patient undergoing primary PCI were screened. All patients (23) who showed a TIMI grade <3 flow at the end of the PCI were enrolled. One milligram was injected slowly over 2 minutes through an infusion catheter distal to the primary stent. TIMI flow grade improved in 87% of patients by at least one category, and the TIMI frame count was reduced from 56 ± 9 frames to 24 ± 4 (p<0.001) [9]. 

Nicardipine has also been shown to be beneficial in a retrospective analysis of 72 patients treated for no reflow with a 99% success rate of restoring TIMI 3 flow. This suggests a class effect from calcium channel blockers in general rather than a specific effect from verapamil [10]. 

### Sodium Nitroprusside

Sodium nitroprusside is hypothesized to prevent and treat no-reflow due to the fact that it is a direct donor of nitric oxide. Nitric oxide has multiple vascular functions including potent vasodilation in the resistance arteriolar circulation and throughout the microcirculation. It also helps to inhibit platelet adhesion and also has anti-inflammatory activity. There is some evidence that nipride is useful in treating no-reflow but has not shown significant benefit in the prevention of no-reflow. 

An initial small study of 20 patients showed significant benefit for treatment of the no-reflow phenomenon. The median injection dose was 200 micrograms given either through the guiding catheter or distally through the angioplasty balloon. Nitroprusside was found to lead to a rapid improvement in both angiographic flow (p<0.01) and blood flow velocity (P<0.01) when compared to the pretreatment angiogram [11]. 

Another small observational treatment study looked at just 11 patients with ST elevation MI who developed the no-reflow phenomenon. Intracoronary nitroprusside in 100mg boluses were given until there was improvement in flow. 82% of the patients improved TIMI flow after nitroprusside (p=0.007) and total frame counts significantly decreased from 36 ± 17 to 16 ± 11 frame counts (p=0.012) [12]. 

When combined with adenosine compared to using adenosine alone for the treatment of no reflow, improvement of TIMI flow grades was higher in the combination group 1.5± 1 vs. 0.8 ± 0.6 (p<.05) [13]. 

For prevention, a randomized trial of 98 patients with STEMI were evenly randomized to receive either 60 micrograms of nitroprusside or placebo delivered into the infarct related artery distal to the occlusion before any angioplasty was done. There was no difference in TIMI frame counts, myocardial blush score or ST segment resolution between the two groups. However, a combined 6 month clinical endpoint of target lesion revascularization, myocardial infarction or death occurred in just 6.3% of the nitroprusside treated group compared to 20% in the placebo (p=.05). This difference was primarily driven by myocardial infarction and TVR but not death. As this was not the primary endpoint of the trial, this outcome can only be considered hypothesis generating. As a whole, this randomized trial was taken to be a negative trial for prevention of no-reflow [14]. However, it is possible that the dose was too low (compared to the higher doses needed in the treatment studies) or the timing was not optimal (given before PCI started). 

### Glycoprotein IIb/IIIa inhibitors

Glycoprotein IIb/IIIa inhibitors are strong antiplatelet agents and should be effective in reducing both epicardial and microvascular thrombus burden. However, there are no convincing randomized trials in using Glycoprotein IIbIIIa inhibitors in the treatment of no-reflow, and therefore their use for *treatment *of no-reflow is not a guideline recommendation. However, a bulk of evidence suggests possible use for the *prevention *of no-reflow. Abciximab has been the most studied of the glycoprotein IIbIIIa inhibitors. 

There is convincing randomized trial data for hard clinical outcomes using IIbIIIa inhibitors in the setting of ST elevation MI [15] and its use in this setting is a Class IIa indication in the current 2011 PCI guidelines [2]. It is possible that one mechanism of benefit is the reduction in the no-reflow phenomenon in the IIbIIIa treated patients. One smaller prospective randomized trial looked at 90 consecutive patients with ST elevation MI. They were randomized to standardized abciximab, intracoronary adenosine given distal to the occlusion or to control. Angiographic no-reflow was only observed in 7% of the abciximab treated group compared to 13% in the adenosine group and 17% in the control group, however due to the small sample size this did not meet statistical significance. Consequently, adverse left ventricular remodeling was seen less (7%) in the abciximab treated patients compared to 30% in both the adenosine and control group (p=.04). At multivariate analysis, the occurrence of no-reflow was an independent predictor of the occurrence of LV remodeling (p=.03, odds ratio 4.9, 95% CI 1.2-20.6) [16]. 

Furthermore, an intracoronary bolus of abciximab has shown decreased microvascular obstruction and infarct size by MRI measurement when compared to intravenous abciximab in a randomized trial of 154 patients. The median infarct size was 15.1% in the intracoronary versus 23.4% in the intravenous group (p=0.01). Similarly, the extent of microvascular obstruction was significantly smaller in intracoronary compared with intravenous abciximab patients (p=0.01) [17]. A larger randomized trial (n=534), the Cicero trial, also looked at intracoronary abciximab versus intravenous abciximab in ST segment elevation MI. The intracoronary bolus dose was 0.25mg/kg. The incidence of myocardial blush grade 2/3 was higher in the intracoronary group than in the intravenous group (76% versus 67%;p=0.022), and the enzymatic infarct size was smaller in the intracoronary group (p=0.008) [18]. However, the largest randomized trial of intracoronary versus intravenous abciximab (AIDA STEMI n=2065) did not show any significant difference in the primary composite outcome of all-cause mortality, recurrent infarction, or new congestive heart failure between the groups. However, the endpoints were lower than expected for this trial suggesting this might not have been a high enough risk group with sufficient thrombus burden to truly test the hypothesis. Furthermore, the intracoronary bolus was shown to significantly decrease the incidence of heart failure at 90 days (2.4% versus 4.1%, p=0.04), and the intracoronary bolus was seen to be more effective in the subgroup of women [19]. 

Intravenous Tirofiban has also showed a preventive effect versus placebo as measured by improved ST segment resolution [20] in the setting of primary PCI. Intravenous Eptifibatide has showed benefit in prevention of no-reflow in the PROTECT TIMI 30 study. In this study, patients randomized to an antithrombin plus Eptifibatide versus an antithrombin alone achieved normal TIMI myocardial perfusion grades more often (57.9% vs. 50.9%, p = 0.048)[21]. This suggests a beneficial class effect from glycoprotein IIbIIIa inhibitors. 

### Nitroglycerin

Intracoronary nitroglycerin has been evaluated for the treatment of no reflow, but it has not shown benefit. This is not surprising due to the pharmacodynamics of nitroglycerin. Nitroglycerin has little impact on arteriolar tone and hence on no-reflow since physiologically it produces little effect in the microvasculature. This is due to the fact that it requires metabolism by the vascular wall to derive its nitric oxide. While the epicardial arteries are able to metabolize the nitroglycerin, the microvascular resistance arterioles are unable to metabolize the nitroglycerin. Therefore, unlike nitroprusside which is a direct nitric oxide donor, it is not thought to have much effect in the no-reflow phenomenon due to microvascular causes. 

In the study previously described for Verapamil treatment of no-reflow [9], Werner *et al* gave intracoronary nitroglycerin injections prior to the verapamil injections in 82% of the patients. TIMI frame counts were measured before and after nitroglycerin was given. No significant difference was seen. Likewise, another small study [22] looking at verapamil treatment for no-reflow also started with a nitroglycerin injection. Again, there was no significant benefit seen to the nitroglycerin treated patients. 

Due to poor results in smaller observational studies and the known moot pathophysiology, there have been no large randomized studies evaluating the use of intracoronary nitroglycerin for no-reflow. 

As stated in the definitions sections, no reflow can also be considered an abrupt onset of TIMI zero flow during an elective percutaneous coronary intervention which could be due to epicardial spasm. This pathophysiology would benefit from intracoronary nitroglycerin. 

## MECHANICAL THERAPY

Besides pharmacotherapy, various mechanical therapies including thrombectomy and distal protection have been used as a method of preventing no reflow. There is no data to *treat *no reflow with any of the mechanical therapies. The largest randomized trial of mechanical aspiration in the setting of ST elevation MI was the TAPAS trial (n=1071, export catheter) [23,24]. This trial utilized myocardial blush grading as the primary endpoint to show that manual aspiration thrombectomy reduced the incidence of no reflow and improved angiographic outcomes. There was also a statistically significant reduction in cardiac death at 1 year (3.6% in the thrombus aspiration group and 6.7% in the conventional PCI group (p = 0.02). On the other hand, as analyzed in the largest randomized trial of rheolytic thrombectomy, the JETSENT trial [25], there was no significant difference in infarct size, TIMI blush grades, or mortality with rheolytic thrombectomy compared to placebo. Therefore, aspiration thrombectomy (but not rheolytic thrombectomy) has gained a class IIa recommendation in the setting of primary PCI in the recent ACC PCI guidelines [2]. Distal protection (with filters or balloon) has also not shown to have any benefit in preventing no reflow in the setting of ST elevation MI [26-28] whereas it has been shown to be beneficial in preventing no reflow in elective saphenous vein PCI [29]. Finally, the idea of direct stenting (rather than predilation with an angioplasty balloon) has been studied in elective and primary PCI as a mechanical measure to prevent no reflow. In elective cases, direct stenting has shown no benefit [30], whereas in primary PCI, one small randomized trial (n=206) showed decrease rates of slow flow or no-reflow as compared to placebo (11.7% vs 26.9%, p=.01) [31]. 

## CONCLUSION

In the setting of a primary PCI, we recommend starting with manual aspiration thrombectomy and then proceeding direct stenting if possible. If no reflow persists in this setting, we recommend bolus injections of 100 micrograms of verapamil, adenosine or nitroprusside with frequent test angiography to look for resolution of no reflow. This can be given through the guiding catheter or more distally via an infusion catheter like the clearway infusion balloon [32] or through an over the wire balloon. If no IIbIIIa inhibitor has been previously used, then we recommend giving one of the IIbIIIa inhibitors as well if no-reflow is persistent despite vasodilatory therapy and the patient is not at high risk of bleeding. 

**Fig. (3):**
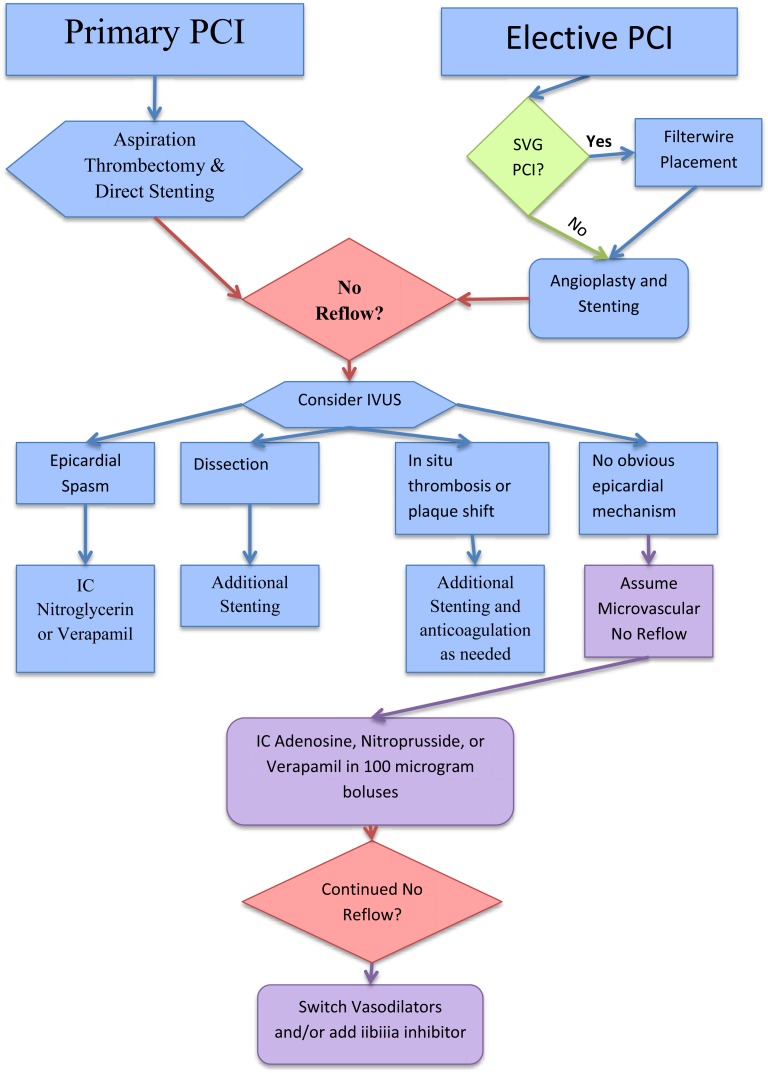
Algorithm for prevention and treatment of no-reflow. PCI=Percutaneous Coronary Intervention, SVG=Saphenous Vein Grafts, IC=Intracoronary

In the setting of elective PCI, the sudden absence of epicardial flow can represent microvascular phenomenon and the we recommend the same dosing of pharmacologic therapy as described. However, there is also the likely incidence of epicardial dissection, epicardial vasospasm or thrombus. Intravascular ultrasound can be helpful in discerning the exact cause of the decreased flow with specific management to follow depending on the cause. In accordance with the 2011 PCI guidelines, we do recommend prophylactic distal protection for elective saphenous vein graft interventions due to the high incidence of embolic debris which potentially could lead to the no-reflow phenomenon (See (Fig. **3**) for prevention and treatment algorithm). 

## Figures and Tables

**Fig. (1):**
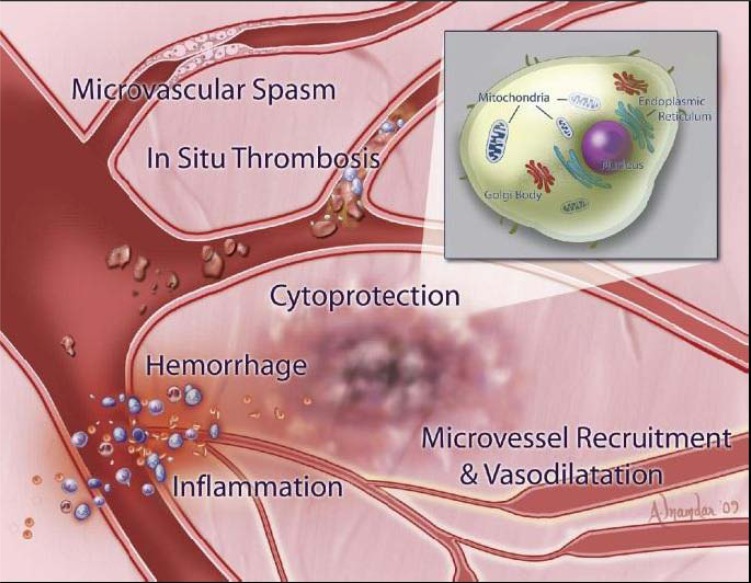
Myocardial targets for pharmacological therapies to prevent no-reflow. The inset depicts a myocardial cell and its subcellular structures involved in cardioprotective pathways. Adapted with permission from Jaffe R, Dick A, and Strauss B. Prevention and Treatment of Microvascular Obstruction-Related Myocardial Injury and Coronary No-Reflow Following Percutaneous Coronary Intervention. A Systematic Approach. J Am Coll Cardiol Intv 2010;3:695-704.

**Fig. (2):**
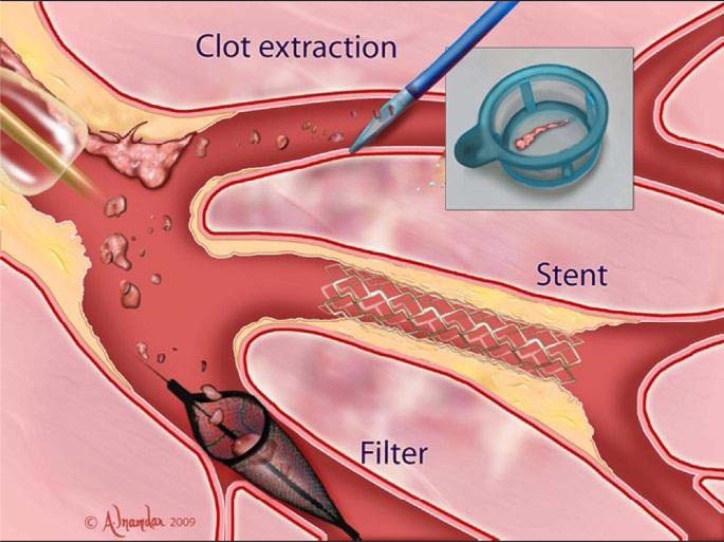
Mechanical strategies to prevent no-reflow. The inset depicts aspirated thrombus. Adapted with permission from Jaffe R, Dick A, and Strauss B. Prevention and Treatment of Microvascular Obstruction-Related Myocardial Injury and Coronary No-Reflow Following Percutaneous Coronary Intervention. A Systematic Approach. J Am Coll Cardiol Intv 2010; 3: 695-704.
